# Occlusion of Regulatory Sequences by Promoter Nucleosomes *In Vivo*

**DOI:** 10.1371/journal.pone.0017521

**Published:** 2011-03-03

**Authors:** Changhui Mao, Christopher R. Brown, Joachim Griesenbeck, Hinrich Boeger

**Affiliations:** 1 Department of Molecular, Cell, and Developmental Biology, University of California Santa Cruz, Santa Cruz, California, United States of America; 2 Department of Biochemistry III, University of Regensburg, Regensburg, Germany; Institute of Genetics and Molecular and Cellular Biology, France

## Abstract

Nucleosomes are believed to inhibit DNA binding by transcription factors. Theoretical attempts to understand the significance of nucleosomes in gene expression and regulation are based upon this assumption. However, nucleosomal inhibition of transcription factor binding to DNA is not complete. Rather, access to nucleosomal DNA depends on a number of factors, including the stereochemistry of transcription factor-DNA interaction, the *in vivo* kinetics of thermal fluctuations in nucleosome structure, and the intracellular concentration of the transcription factor. *In vitro* binding studies must therefore be complemented with *in vivo* measurements. The inducible *PHO5* promoter of yeast has played a prominent role in this discussion. It bears two binding sites for the transcriptional activator Pho4, which at the repressed promoter are positioned within a nucleosome and in the linker region between two nucleosomes, respectively. Earlier studies suggested that the nucleosomal binding site is inaccessible to Pho4 binding in the absence of chromatin remodeling. However, this notion has been challenged by several recent reports. We therefore have reanalyzed transcription factor binding to the *PHO5* promoter *in vivo*, using ‘chromatin endogenous cleavage’ (ChEC). Our results unambiguously demonstrate that nucleosomes effectively interfere with the binding of Pho4 and other critical transcription factors to regulatory sequences of the *PHO5* promoter. Our data furthermore suggest that Pho4 recruits the TATA box binding protein to the *PHO5* promoter.

## Introduction

*In vitro* studies indicated that the wrapping of DNA in nucleosomes limits the accessibility of core particle DNA to nucleases and transcription factors and interferes with the initiation of transcription [1], [2], [3]. This conclusion provides the basis for theories regarding the gene-regulatory function of chromatin structure, and has sparked great interest in the mechanism of nucleosome positioning [4], [5], [6], [7], and the kinetics of nucleosome transactions *in vivo* [8].

However, the occlusion of binding sites by nucleosomes is not complete. A small number of DNA binding proteins appears to bind wrapped nucleosomal DNA, albeit at reduced affinity, as long as their recognition sequence is rotationally properly positioned [9]. Most transcription factors probably depend on the spontaneous unwrapping of nucleosomal DNA to access interior sequences of the core particle [2]. This requires sufficiently high concentrations of the transcription factor to overcome the fast rewrapping kinetics of nucleosomal DNA [10]. Clustering of binding sites within the nucleosome core particle can lead to cooperative binding in the absence of direct interactions between the transcription factors (indirect cooperativity) [11]. Furthermore, it has been argued that histone modifications affect the extent of DNA wrapping about the histone octamer [12], [13].

Because of these and other mitigating factors, *in vitro* binding studies, which have mostly been performed on nucleosomes reconstituted *in vitro* on artificial DNA sequences, need to be complemented by *in vivo* experiments when considering the effect of specific nucleosomes on transcription factor binding [14]. *In vivo* binding studies have focused on a small number of biological models. The inducible *PHO5* promoter of yeast (*Saccharomyces cerevisiae*) has served as a prominent paradigm in this discussion [15]. *PHO5*, which encodes a secreted acidic phosphatase, is induced in response to phosphate starvation. The *PHO5* promoter contains three regulatory sequence elements, two upstream activation sequences, UASp1 and UASp2, and a TATA box. Under repressing conditions (high phosphate media), the promoter is characterized by nucleosomes in defined positions, with UASp1 exposed in the linker region between the two nucleosome core particles, N-2 and N-3, and UASp2 positioned close to the center of core particle N-2; the TATA box is wrapped in core particle N-1 [16]. Under activating conditions (media with little or no phosphate), the transcriptional activator Pho4, a helix-loop-helix DNA binding protein, enters the nucleus and binds together with the homeodomain factor Pho2 at both upstream activating sequences. The activation domain of Pho4 is required for the depletion of promoter nucleosomes and the activation of *PHO5* transcription [8], [17].

Nucleosome N-2 isolated from native yeast chromatin was found to prevent the binding of Pho4 and Pho2 at UASp2 [18]. A classic experiment used dimethylsulfate (DMS) footprinting *in vivo* to show that Pho4, when deprived of its activation domain, binds at UASp1, but not UASp2 [19]. Consistently, UASp1 residues were methylated at a faster rate than UASp2 residues upon activation of *PHO5* by Pho4 fused to a DNA methyltransferase [20]. Thus *in vitro* and *in vivo* binding experiments suggested occlusion of the Pho4 binding site at UASp2 by nucleosome N-2.

However, doubts remain. The pattern of a potential Pho4-DMS footprint on nucleosomal DNA is unknown. The absence of a recognizable pattern does therefore not exclude the possibility of Pho4 binding to the nucleosome. Free methyltransferase methylates nucleosomal DNA at a slower rate than naked DNA [20]. Slower methylation kinetics may therefore reflect the presence of a nucleosome rather than the inability of Pho4-DMS to access its binding site at UASp2. Furthermore, recent studies provided evidence, either by DMS footprinting or chromatin immunoprecipitation (ChIP), for binding of Pho4 at UASp2 in the absence of apparent chromatin remodeling, which was inhibited by deletion of the histone chaperone gene *ASF1* [21], [22], [23]. These results suggested that Pho4 binding to DNA was uninhibited by nucleosome formation. On the basis of these conclusions, the binding of Pho4 and Pho2 at UASp2 has been construed as an *in vivo* example for indirect cooperative binding of transcription factors to nucleosomal DNA [24].

Because of its significance for theoretical attempts to understand the role of chromatin structure in gene expression and regulation [8], [25], we reanalyzed Pho4 binding, and investigated the binding of TBP and Pho2 at the *PHO5* promoter in wild type cells and various mutants by ‘chromatin endogenous cleavage’ (ChEC). This approach allows one to monitor binding of transcription factors to their recognition elements by measuring the frequency of binding-site dependent DNA cleavage, after *in vivo* crosslinking, by micrococcal nuclease that was linked to the transcription factor [26]. This approach allowed for quantitative measurements of high molecular specificity, sufficient spatial resolution, as well as low background, but was superior to other previously used methods mostly because the absence of signal was more easily interpretable (see Discussion).

## Results

### *PHO5* promoter cleavage by Pho4-MNase

**Figure 1:**
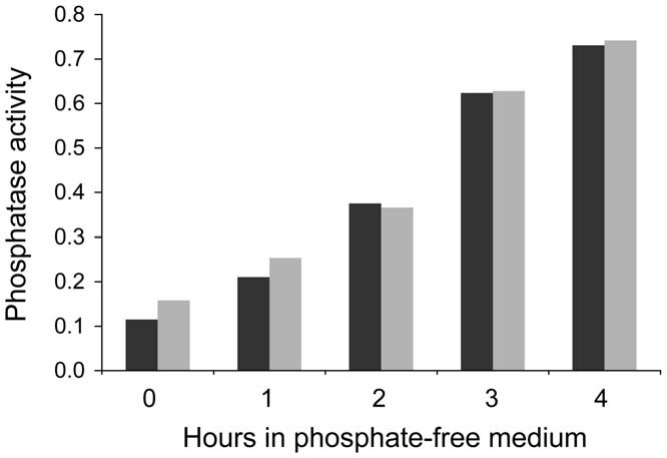
Linkage of MNase to Pho4 does not interfere with *PHO5* activation. Acidic phosphatase activities were measured after 0, 1, 2, 3, and 4 hours of culturing cells in phosphate-free medium. Phosphatase activities for cells expressing the Pho4 wild type protein and Pho4-MNase are indicated by gray and black bars, respectively. Phosphatase activity is given in arbitrary units normalized to cell density.

To analyze Pho4 binding at the *PHO5* promoter, we generated strains that express micrococcal nuclease linked to the C-terminus of the transcription factor (Pho4-MNase). Phosphatase assays indicated that the fusion protein was equally effective in activating *PHO5* as the wild type Pho4 protein (Fig. 1).

**Figure 2:**
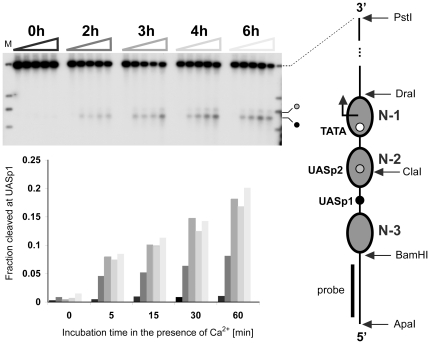
Pho4-MNase cleaves *PHO5* promoter DNA close to known Pho4 binding sites. ChEC analysis of Pho4 binding at the wild type *PHO5* promoter. Cells were cultured in phosphate-free medium for 0, 2, 3, 4, and 6 hours before analysis. For each induction time point, aliquots of extract prepared after in vivo cross-linking were incubated in the presence of Ca^2+^ ions for 0, 5, 15, 30, and 60 minutes (triangles above autoradiographs). The DNA was isolated, digested with PstI and ApaI, fractionated by agarose gel electrophoresis, blotted and hybridized with a ^32^P-labeled DNA probe complementary to sequences upstream of the *PHO5* promoter. Autoradiography of the Southern blot is shown on top. The graphic on the right shows the nucleosome configuration of the repressed *PHO5* promoter [16], [29], where nucleosomes and regulatory sequence elements are indicated by gray ovals and small circles, respectively. The relative frequency of cleavage at UASp1, which provides a measure for Pho4 binding at UASp1, is indicated on the ordinate of the histogram (bottom). Different gray tones correspond to different induction times (see triangles above autoradiography). Numbers on the abscissa indicate incubation times (in minutes) in the presence of calcium ions. The similarity of cleavage kinetics for induction time points 3, 4, and 6 hours suggests that Pho4 reached its binding equilibrium at UASp1 between 2 and 3 hours after transfer of cells into phosphate-free medium. Marker bands (lane M) indicate (from top to bottom) restriction sites for PstI (3′-end of *PHO5* gene), DraI (beginning of open reading frame), ClaI (UASp2), and BamHI (5′-edge of nucleosome N-3).

Cells expressing Pho4-MNase were briefly treated with formaldehyde to cross-link promoter-bound proteins and DNA, either before or at different times after transfer into phosphate-free medium. Extracts prepared from cross-linked cells were incubated for various amounts of time in the presence of Ca^2+^ ions to activate micrococcal nuclease. To determine cleavage frequencies, isolated DNA was digested with restriction enzymes to release a 3 kb fragment encompassing the *PHO5* gene, fractionated by gel electrophoresis, blotted and hybridized with a radiolabeled DNA probe that recognizes sequences upstream of the *PHO5* promoter (Fig. 2). As expected, *PHO5* DNA isolated from induced cells was cleaved at two sites, close to UASp1 and UASp2. Both sites most certainly represent a cluster of closely spaced cutting events, as micrococcal nuclease lacks sequence specificity. There was little or no cleavage of *PHO5* DNA isolated from repressed cells. Cleavage frequencies for samples taken at 3, 4 and 6 hours after induction were virtually identical (Fig. 2), suggesting that Pho4 reached binding equilibrium at UASp1 between 2 and 3 hours after induction. The slow approach toward binding equilibrium may explain, in part, the slow kinetics of *PHO5* induction and promoter nucleosome loss [27]. An even slower approach toward binding equilibrium was previously observed by ChIP [28]. The discrepancy may be attributable to the lack of resolution in the ChIP experiments, which did not allow for distinguishing between binding at UASp1 and UASp2.

**Figure 3:**
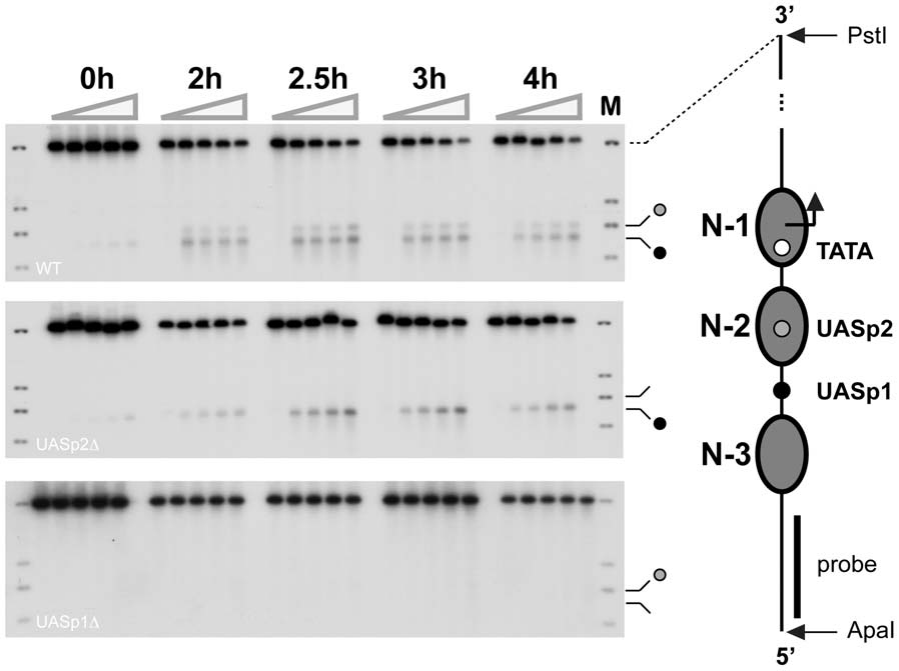
Effect of UASp mutations on Pho4 binding at the *PHO5* promoter. ChEC analysis of Pho4 binding at the *PHO5* promoter. Cells were cultured in phosphate-free medium for 0, 2, 2.5, 3, and 4 hours before analysis. Cell extracts for each induction time point were incubated in the presence of Ca^2+^ ions for 0, 5, 15, 30, and 60 minutes (gray triangles above autoradiographs). The DNA was isolated, digested with PstI and ApaI, fractionated by agarose gel electrophoresis, blotted and hybridized with a radioactively labeled DNA probe spanning sequences upstream of the *PHO5* promoter. A diagram of the repressed *PHO5* promoter chromatin structure is shown to the right of the autoradiographs. Gray ovals represent nucleosome core particles. Positions of the TATA-box, UASp2, and UASp1 are indicated by a white, gray and black dot, respectively. A black bar indicates the position of the probe used for indirect end-labeling. Autoradiographs on the top, middle and bottom show Pho4 binding in *PHO5* wild type cells, UASp2Δ cells, and UASp1Δ cells, respectively. Bands in the marker lane (M) indicate, from top to bottom, positions of PstI, DraI, ClaI and BamHI restriction sites, respectively. Pho4 binding at the *PHO8* promoter was unimpaired in UASp1Δ cells (data not shown).

The restriction of cleavage to sites close to UASp1 and UASp2 suggested that cutting was due to sequence-specific binding of Pho4-MNase at the promoter. To test this conjecture, we mutated the Pho4 binding site at UASp2. The mutation selectively abolished cleavage at UASp2 but not UASp1 (Fig. 3), indicating that cleavage close to UASp2 was due to Pho4 binding at UASp2, and not due to Pho4 binding at UASp1 or cross-linking of Pho4-MNase that did not interact with the *PHO5* promoter in a sequence-specific manner.

### Mutation of UASp1 prevents Pho4 binding at UASp2

**Figure 4:**
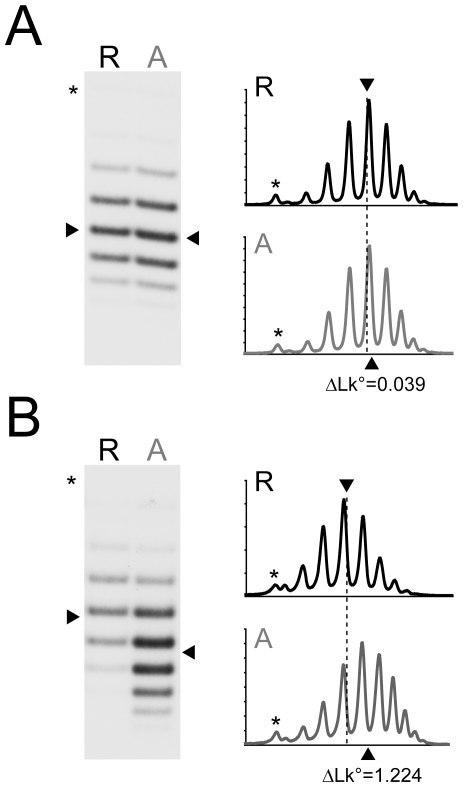
Effect of UASp mutations on *PHO5* promoter nucleosome loss. Nucleosome loss at the *PHO5* promoter was measured by topology analysis [29], in UASp1 and UASp2 deletion mutants. Gene circles were formed *in vivo* by site-specific recombination of the *PHO5* locus [29]. Gene circle topoisomers were fractionated by chloroquine gel electrophoresis, blotted and hybridized with a ^32^P-labeled DNA probe spanning the *PHO5* gene. (**A**) Topoisomer distributions of *PHO80* (R) and *pho80*Δ (A) *PHO5* gene circles isolated from UASp1Δ cells. Pho80 is an inhibitor of the PHO signaling pathway. In its absence Pho4 is constitutively nuclear. (**B**) Topoisomer distributions of *PHO80* (R) and *pho80*Δ (A) *PHO5* gene circles isolated from UASp2Δ cells. The intensity profiles of distributions are shown on the right next to the autoradiographs. Distribution centers are indicated by arrowheads. The center of the topoisomer distribution for the repressed gene is additionally marked by a dashed line in the intensity profiles. Numbers indicate the linking difference, ΔLk°, between distribution means (arrowheads), which corresponds to the average number of nucleosomes lost from the activated promoter [29]. Wild type *PHO5* gene circles have a linking difference of 1.85 [8], [29]. Positions of nicked circles are indicated by (*).

To analyze effect of UASp mutations on the chromatin remodeling on the *PHO5* promoter, we employed strains that allow for the formation of *PHO5* gene circles *in vivo* and subsequent analysis of chromatin remodeling by topology analysis [29]. We isolated gene circle topoisomers from UASp1 and UASp2 mutant strains, and resolved topoisomers by agarose gel electrophoresis. Consistent with earlier nuclease accessibility measurements at nucleosome N-2 [30], mutation of UASp1 completely abolished remodeling of *PHO5* promoter chromatin, as indicated by virtually identical gene circle topoisomer distributions between induced and non-induced cells (Fig. 4A). In contrast, mutation of UASp2 allowed for chromatin remodeling, although remodeling was less effective than in promoter wild type cells (Fig. 4B).

To assess whether nucleosome N-2 interferes with Pho4 binding at UASp2, we repeated our ChEC analysis in a UASp1 mutant. If nucleosome N-2 does not interfere with Pho4 binding at UASp2, mutation of UASp1 should selectively abolish cleavage at UASp1, but not UASp2. In contrast, if Pho4 binding at UASp2 is inhibited by nucleosome N-2, mutation of UASp1 is expected to abolish cleavage at both UASp1 and UASp2. Our results bore out the latter expectation (Fig. 3). The simplest interpretation of this result is that UASp2 is inaccessible to Pho4, unless nucleosome N-2 is removed due to Pho4 binding at UASp1 and recruitment of chromatin remodeling or other activities.

### Mutation of UASp1 prevents Pho2 binding at UASp2

**Figure 5:**
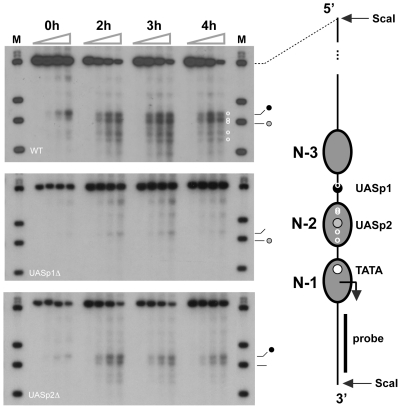
Effect of UASp mutations on Pho2 binding at the *PHO5* promoter. ChEC analysis of Pho4 binding at the *PHO5* promoter. Cells were cultured in phosphate-free medium for 0, 2, 3, and 4 hours before analysis. Cell extracts for each induction time point were incubated in the presence of Ca^2+^ ions for 0, 5, 15, 30, and 60 minutes (gray triangles above autoradiographs). The DNA was isolated, digested with ScaI, fractionated by agarose gel electrophoresis, blotted and hybridized with a radioactively labeled DNA probe spanning sequences within the *PHO5* open reading frame. A diagram of the repressed *PHO5* promoter chromatin structure is shown to the right of the autoradiographs. Gray ovals represent nucleosome core particles. Positions of the TATA-box, UASp2, and UASp1 are indicated by a white, gray and black dot, respectively. White circles indicate positions of Pho2 binding sites mapped by DNase I footprinting *in vitro* and corresponding Pho2-MNase cleavage pattern (autoradiography). A black bar indicates the position of the probe used for indirect end-labeling. Autoradiographs on the top, middle and bottom show Pho2p binding in *PHO5* wild type cells, UASp1Δ cells, and UASp2Δ cells, respectively. Bands in the marker lane (M) indicate, from top to bottom, positions of ScaI, BamHI, ClaI and DraI restriction sites, respectively. (The ScaI/ScaI fragments of the samples in the bottom autoradiograph migrated slower than the Sca/ScaI fragment of the marker, because the Pho2-MNase expressing strain was derived from a *PHO5* gene circle strain, which contains an RS element between nucleosome N-3 and the 5′ ScaI site [29], whereas the marker was prepared from a strain lacking this insertion. This did not affect the positioning of Pho2-MNase cleavage sites relative to the ClaI and DraI marker bands.

Multiple Pho2-binding sites have been detected by DNase I footprinting *in vitro* at the *PHO5* promoter, including one site juxtaposed to UASp1, and four sites occupied by nucleosome N-2 under repressing conditions [31]. A corresponding cleavage pattern of *PHO5* DNA was observed for cells expressing Pho2-MNase after induction (Fig. 5). Mutation of UASp1 abolished cleavage by Pho2-MNase, except at UASp1, indicating that nucleosome N-2 interfered with Pho2 binding. In contrast, mutation of UASp2 allowed for Pho2 binding at N-2 sequences, albeit with reduced apparent affinity, consistent with the increased promoter nucleosome occupancy in the induced UASp2 mutant compared to wild type (Fig. 4B).

### TBP binding at the *PHO5* promoter coincides with transcriptional activation of *PHO5*

**Figure 6:**
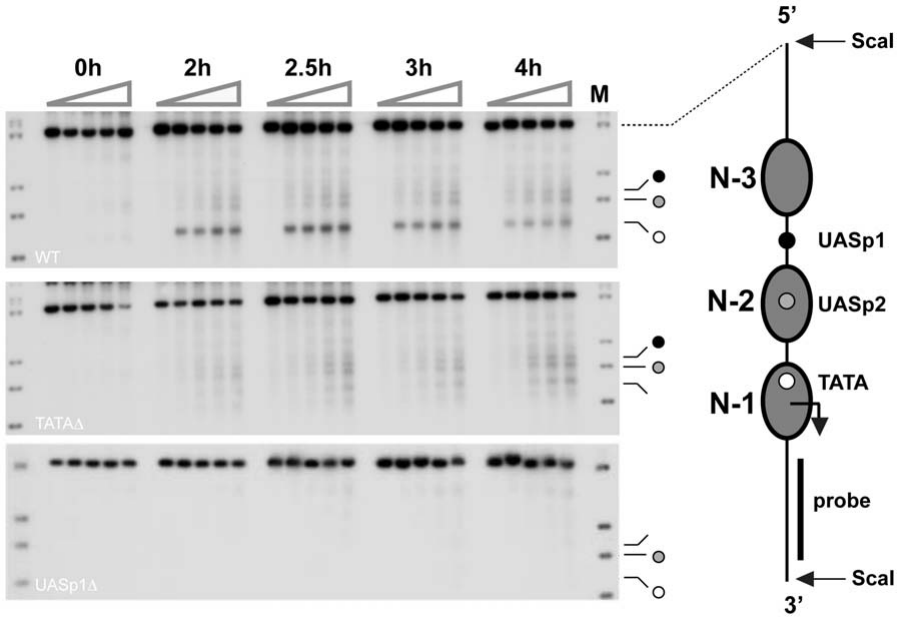
TBP binding at the *PHO5* promoter requires binding of Pho4 at UASp1. ChEC analysis of TBP-MNase cleavage of *PHO5* promoter DNA in the *PHO5* wild type, and strains bearing mutations either in the TATA box or UASp1 of the *PHO5* promoter. Autoradiographs show cleavage of *PHO5* promoter DNA for *PHO5* wild type (top), a TATA box mutant (middle), and a UASp1Δ mutant (bottom), respectively. Bands in the marker lane (M) indicate, from top to bottom, ScaI, BamHI, ClaI, and DraI cleavage, respectively.

Does nucleosome N-1 occlude the promoter's TATA box? To address this question, we investigated the cutting of *PHO5* promoter DNA by micrococcal nuclease linked to the TATA box binding protein (TBP-MNase). Upon induction, a distinct cleavage pattern was observed, with strong cutting at the TATA box, and weaker cutting at UASp1 and UASp2 (Fig. 6). In contrast, little or no cleavage was observed under repressing conditions (Fig. 6), consistent with the notion that TBP binding at the *PHO5* TATA box requires prior removal of nucleosome N-1.

Cleavage at all three promoter sites was abolished in a UASp1 mutant, indicating that cutting, including cuts at the non-nucleosomal UASp1, required binding of Pho4 to the promoter (Fig. 6). While cleavage at UASp2 and the TATA box might have been due to loss of nucleosomes from positions N-1 and N-2 and nonspecific DNA binding by TBP-MNase, Pho4-dependent cleavage at UASp1 either indicated recruitment of TBP-MNase by the promoter-bound activator, or interactions between the core promoter and upstream activating sequences. To distinguish between these two possibilities, we investigated cutting by TBP-MNase in a TATA box mutant. If cleavage at upstream activating sequences was entirely due to TBP-MNase binding at the TATA box and looping of DNA between the TATA box and activator binding sites, mutation of the TATA box should diminish cleavage at all three promoter sites. In contrast, we found that mutation of the TATA box diminished cleavage at the core promoter, but not at UASp1 and UASp2 (Fig. 6).

### Pho4 and Pho2 bind cooperatively at the *PHO5* promoter *in vivo*

*In vitro* binding studies showed that Pho4 binds cooperatively with Pho2 at UASp1 [31]. To determine the significance of this cooperativity for DNA binding *in vivo*, which depends on the intracellular concentration of both transcription factors, we investigated cleavage of *PHO5* promoter DNA by Pho4-MNase and Pho2-MNase in *pho2*Δ and *pho4* Δ strains, respectively.

**Figure 7:**
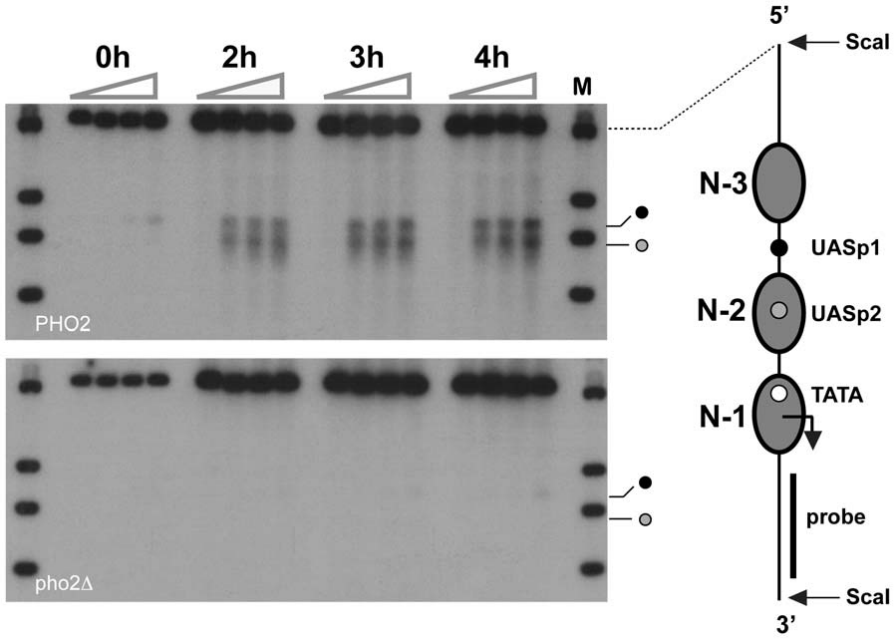
Deletion of PHO2 abolishes Pho4 binding at the *PHO5* promoter. ChEC analysis of Pho4 binding at *PHO5* promoter in wild type and *pho2*Δ cells. *PHO2* wild type (PHO2) and *pho2*Δ cells (pho2Δ) cells expressing Pho4-MNase were cultured in phosphate-free media for 0, 2, 3, and 4 hours. Cell extracts for each induction time point were incubated in the presence of Ca^2+^ ions for 0, 10, 30, and 60 minutes (triangles above autoradiograph). For marker bands see legend to Fig. 3.

**Figure 8:**
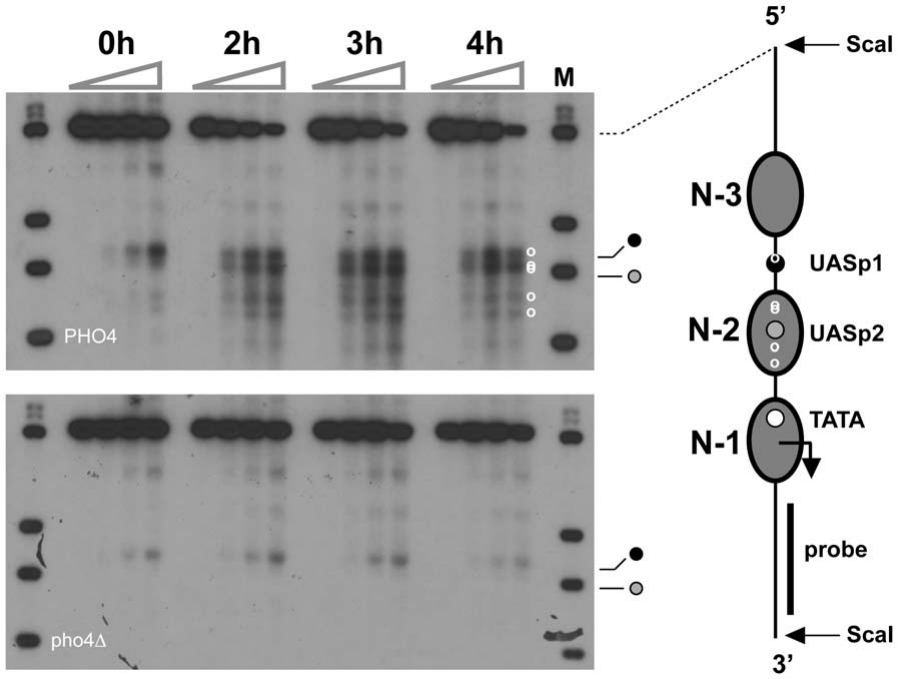
Pho4 stabilizes Pho2 binding at linker DNA. ChEC analysis of Pho2 binding at the *PHO5* promoter. Pho4 wild type (PHO4) and deletion (pho4Δ) cells of Pho2-MNase strains were treated as described in the legend to Fig. 5. Top autoradiograph (analysis of *PHO4* wild type strain) is identical to the top autoradiograph of Fig. 5. Bottom autoradiograph shows *PHO5* promoter cleavage by Pho2-MNase for *pho4*Δ cells.

Deletion of *PHO2* abolished cleavage of the activated promoter DNA by Pho4-MNase (Fig. 7). This suggested that the cooperativity between Pho4 and Pho2 observed *in vitro* is essential for Pho4 binding at UASp1 *in vivo*, consistent with the absolute requirement of Pho2 for transcriptional activation of *PHO5*. Deletion of *PHO4* abolished cleavage of the activated promoter by Pho2-MNase at N-2 sequences, as expected, and decreased cleavage at UASp1 (Fig. 8). The latter observation suggested that the direct interaction between Pho4 and Pho2 also stabilized Pho2 binding at UASp1. Consistent with an earlier finding that Pho2 recruits the histone acetyltransferase NuA4 to the repressed *PHO5* promoter [32], our data suggested weak binding of Pho2 at UASp1 in the absence of Pho4.

## Discussion

Our data demonstrate that, *in vivo*, *PHO5* promoter sequences occupied by nucleosomes remain largely inaccessible to the critical transcription factors Pho4, Pho2, and TBP, unless nucleosomal inhibition is relieved upon Pho4 binding at the linker-positioned UASp1 (Fig. 3, 5, 6).

Occlusion of UASp2 by nucleosome N-2 is consistent with the crystal structures of the nucleosome, and the basic helix-loop-helix domain of Pho4 bound at UASp2 [33], [34]. The Pho4 homodimer contacts its binding sequence in the major groove on two opposite faces of the DNA, with the helix-loop-helix domain on one face and the remainder of the protein, including its activation domain, on the other face of the DNA [34]. This stereochemistry precludes binding to DNA adhering to a surface. It must be assumed, therefore, that binding of Pho4 to UASp2 requires unwrapping of the DNA from the histone octamer of nucleosome N-2. Consistently, the DNA in the crystal structure of Pho4-UASp2 complex is unbent with a helical repeat of 10.77 base pairs per turn [34], close to that of free DNA under physiological conditions [35], but different from the average helical repeat of 10.2 base pairs per turn for the nucleosome core particle [9], [33].

Contrary to recent suggestions [21], [22], [23], our data indicate that the physiological concentration of Pho4 is insufficient to capture transiently unfolded states of the nucleosome due to either thermal fluctuations in DNA-histone interactions [2], or constitutive enzymatic remodeling. The effect of UASp1 deletion on *PHO5* expression and chromatin remodeling can be suppressed by overexpression of Pho4 [19], [36]. Suppression may result from non-specific binding of Pho4 at linker DNA, or direct access to UASp2. The latter possibility does not contradict our conclusions, as Pho4 concentrations above the physiological level may allow for efficient capturing of short-lived unfolded nucleosome states [2].

Like Pho4, Pho2 and the TATA box binding protein are efficiently excluded from their binding sequences when the latter are wrapped in a nucleosome (Fig. 5, 6). Under repressing conditions Pho2 occupied its binding site close to UASp1, but no binding was detectable at N-2 sequences (Fig. 5). The latter cannot be explained by the assumption that Pho2 binding at N-2 sequences required Pho4 binding at UASp2, because Pho2 could access N-2 sequences under activating conditions in UASp2 mutant cells (Fig. 5). Although positioned close to the entry site to nucleosome core particle N-1 [29], [37], the TATA box was not measurably occupied by TBP under repressing conditions (Fig. 6), suggesting that nucleosome N-1 interfered with TBP binding at the TATA box. This conclusion is consistent with other experimental findings. Based on the assumption that transcriptional activation of *PHO5* requires removal of nucleosome N-1, it has been possible to explain the quantitative relationship between expression level, promoter nucleosome occupancy, and the magnitude of steady-state fluctuations in gene expression at the single cell level [8]. Furthermore, depletion of nucleosomes *in vivo* promotes activator-independent transcription of *PHO5* and initiates transcription from cryptic promoters [38], [39].

Most recently, it has been suggested that absence of Asf1 allows for binding of Pho4 to a partially unfolded core particle rather than a fully wrapped nucleosome [22], [23]. As pointed out above, for structural reasons it must be assumed that Pho4 binding at UASp2 requires at least partial unfolding of nucleosome N-2. The partially unfolded structure was assumed to be conducive to Pho4 binding at UASp2 and yet proved resistant to nuclease digestion [21], [23]. No attempt was made to explain the apparent contradiction. A first attempt to prove the existence of the altered nucleosome fell short because the resolution afforded by ChIP of sonicated chromatin was insufficient to distinguish between Pho4 binding at UASp1 and UASp2 [22]. Instead, it was argued that immunoprecipitation of *PHO5* promoter DNA from chromatin preparations of *asf1*Δ cells with an anti-Pho4 antibody was mostly due to Pho4 binding at UASp2 rather than UASp1, since Pho4 binds (naked) UASp2 with higher affinity than UASp1 [22], [23]. However, this argument presupposed the validity of what the experiment needed to show, that Pho4 bound with equal affinity to naked and nucleosomal UASp2. In a second attempt, micrococcal nuclease-generated nucleosome core particles were sequentially immunoprecipitated, firstly with an anti-Pho4 antibody and secondly with an anti-histone H3 antibody [23]. Nucleosome N-2 DNA was pulled down more efficiently in chromatin preparations from *asf1*Δ cells than wild type cells, suggesting that Pho4 and histones bound to the same sequences simultaneously [23]. However, the difference in immunoprecipitation efficiency between wild type and *asf1*Δ cells may be attributable to the histone antibody alone. In chromatin preparation from wild type cells, Pho4-bound sequences must have been mostly naked and thus degraded by micrococcal nuclease, whereas UASp2 sequences that were precipitated non-specifically in the first immunoprecipitation step were mostly nucleosomal in preparations from *asf1*Δ cells, and thus efficiently precipitated by the histone antibody in the second step. These experiments did not, therefore, provide a compelling argument for simultaneous binding of histones and Pho4 to N-2 sequences. We have analyzed Pho4 binding at UASp2 in a UASp1 *asf1*Δ mutant to address the possibility that nucleosome N-2 is altered rather than removed in *asf1*Δ cells upon *PHO5* induction. We could find no evidence for this possibility. Our results were indistinguishable from those obtained with *ASF1* wild type cells that bore a mutated UASp1 (data not shown).

Two objections may be raised against the naïve interpretation of ChEC data. First, the fusion of Pho4 to micrococcal nuclease prevented binding of Pho4 to nucleosome N-2. This appears unlikely because micrococcal nuclease did not measurably interfere with *PHO5* activation (Fig. 1). Second, N-2 may not have prevented Pho4 binding at UASp2, but cleavage of promoter DNA by micrococcal nuclease. The question of whether the absence of signal may be interpreted as absence of binding equally applies to other methods for detecting the binding of transcription factors to DNA (see Introduction). For ChEC, the question may be answered affirmatively. Binding of Pho4-MNase at the nucleosomal UASp2, close to the dyad axis of the N-2 nucleosome core particle, would position the nuclease in close proximity to the nucleosomal linkers. Since micrococcal nuclease does not rely on specific DNA sequences for cleavage and effectively cleaves linker DNA when tethered to nucleosomes by linkage to the C-terminus of histones [26], [40], it must be assumed that absence of cleavage indicates absence of transcription factor binding. This is not necessarily the case for DNA methylation and DMS footprinting assays, as both methods rely on modification of DNA occupied by core particle N-2 for detection of Pho4 binding at UASp2.

Different conclusions regarding the ability of Pho4 to access its binding site within nucleosome N-2 are most likely attributable to the different methods used to prevent the loss of promoter nucleosomes under inducing conditions - mutation of the Pho4 binding site at UASp1 in this and other studies [19], [41], and deletion of *ASF1* in recent reports [21], [22], [23]. The effect of the UASp1 mutation on *PHO5* regulation is undoubtedly direct. This may not be true for the *asf1*Δ mutant. Cells that lack Asf1 function exhibit a wide range of defects in gene expression, including the expression of histone genes [42], [43]. Furthermore, *ASF1* was found to be required for *PHO5* chromatin remodeling at intermediate but not low phosphate concentrations [44]. This may have rendered the outcome of induction experiments in *asf1*Δ cells susceptible to small irregularities in the phosphate concentration of the media [21], [22], [44].

Our ChEC results provided evidence for recruitment of TBP to the *PHO5* promoter by Pho4 *in vivo* (Fig. 6). This result is consistent with earlier demonstrations of Pho4 and TBP interaction *in vitro* [45]. The *in vivo* interaction does not have to be direct but may be mediated by other factors such as TFIIB, which was also found to interact with Pho4 *in vitro* [46]. Cleavage of *PHO5* promoter DNA by TBP-MNase at upstream activating sequences may be due to TBP-MNase bound at the TATA box and loop formation between the core promoter and upstream activating sequences, rather than recruitment by Pho4. Two observations argue against this possibility. Loop formation should promote cleavage of core promoter sequences by Pho4-MNase. No such cleavage was observed (Fig. 7). If cleavage at upstream activating sequences by TBP-MNase were due to loop formation, mutation of the TATA box should diminish the frequency of cleavage at the upstream activating sequences. However, cleavage was diminished only at the TATA box, and not at the upstream activating sequences (Fig. 6). Activator interactions with other proteins are promiscuous by nature. Their relevance for the regulatory mechanism is therefore uncertain. However, the suggested interaction between TBP and Pho4 may be physiologically significant. Analysis of the steady-state fluctuations in *PHO5* promoter-controlled gene expression indicated that the Pho4 activator stimulates the rate of transcription machinery assembly after promoter nucleosome removal [8].

## Materials and Methods

### Plasmids and Strains

The *PHO5* UASp1, UASp2 and TATA box mutations were described previously [8], [29]. Pho4 and Pho2 deletion plasmids pCM90.1 and pCM115.1 were constructed by replacing the *PHO4* or *PHO2* open reading frame with a 1.1 kb *URA3* gene.

*PHO4* and *PHO2*-MNase fusion strains YR22 and YR3 were derived from strains NOY505 and yM2.1 [29], respectively, as previously described [40]. Strain y1185, expressing TBP-MNase, was reported earlier [40]. *PHO5Δ*::*URA3* strains yC01.2, yC11.3, and yC131.8 were constructed by replacing *PHO5* in YR22, y1185, and YR3 using plasmid pM51.1 as described previously [29]. Strains yC03.1 and yC15.1, containing *PHO5* UASp1 and UASp2 mutations in addition to Pho4-MNase, were derived from yC01.2 by homologous recombination with plasmids pCM46.9 and pCM65.1, respectively. Similarly, strain yC16.1, expressing TBP-MNase, was derived from yC11.3 with plasmid pCM46.9. Strains yC159.3, yC138.6, and yC139.5, expressing *PHO2*-MNase, were derived from yC131.8 with plasmids pM50.1, pCM46.9, and pCM47.8 bearing wild type *PHO5*, *PHO5* UASp1 mutation, and *PHO5[GC]* UASp2 mutation. *PHO5* TATA box mutation strain yC22.2, expressing TBP-MNase, was derived from yC11.3 with plasmid pCM74.3. Strain yC162.1, a *PHO4Δ*::*URA3* strain expressing *PHO2*-MNase, was derived from yC159.3 with plasmid pCM90.1. Strain yC132.1, a *PHO2Δ*::*URA3* strain expressing Pho4-MNase, was derived from YR22 with plasmid pCM115.1. A list of all strains used is provided in Table 1. All yeast transformations were performed using the lithium acetate method.

**Table 1:** Yeast strains list.

Name	Parent	Genotype	Source
NOY505		mata; ade2-1; ura3-1; trp1-1; leu2-3,112; his3-11; can1-100	Merz et al. 2008
YR22	NOY505	mata; ade2-1; ura3-1; trp1-1; leu2-3,112; his3-11; can1-100; PHO4-MNase-3xHA KanMX6	This study
y1185	NOY505	mata; ade2-1; ura3-1; trp1-1; leu2-3,112; his3-11; can1-100; SPT15-MNase-3xHA KanMX6	Merz et al. 2008
YR3	yM2.1	matα; his3-11; his3-15; leu2-3; leu2-112; canR; ura3Δ5; PHO5[GC]; PHO2-MNase-3xHA KanMX6	This study
yC01.2	YR22	mata; ade2-1; ura3-1; trp1-1; leu2-3,112; his3-11; can1-100; PHO4-MNase-3xHA KanMX6; PHO5Δ::URA3	This study
yC03.1	yC01.2	mata; ade2-1; ura3-1; trp1-1; leu2-3,112; his3-11; can1-100; PHO4-MNase-3xHA KanMX6; PHO5::UASp1mut	This study
yC11.3	y1185	mata; ade2-1; ura3-1; trp1-1; leu2-3,112; his3-11; can1-100; SPT15-MNase-3xHA KanMX6; PHO5Δ::URA3	This study
yC15.1	yC01.2	mata; ade2-1; ura3-1; trp1-1; leu2-3,112; his3-11; can1-100; PHO4-MNase-3xHA KanMX6; PHO5::UASp2mut	This study
yC16.1	yC11.3	mata; ade2-1; ura3-1; trp1-1; leu2-3,112; his3-11; can1-100; SPT15-MNase-3xHA KanMX6; PHO5::UASp1mut	This study
yC18.2	yM17.3	matα; his3-11; his3-15; leu2-3; leu2-112; canR; ura3Δ5; pho5[GC, <TATA>]:UASP2mut; pho80Δ::HIS3	Mao et al. 2010
yC19.3	yM17.3	matα; his3-11; his3-15; leu2-3; leu2-112; canR; ura3Δ5; pho5[GC, <TATA>]:UASP1mut pho80Δ::HIS3	Mao et al. 2010
yC22.2	yC11.3	mata; ade2-1; ura3-1; trp1-1; leu2-3,112; his3-11; can1-100; SPT15-MNase-3xHA KanMX6; PHO5::TATAmut	This study
yC23.2	yM1.12	matα; his3-11; his3-15; leu2-3; leu2-112; canR; ura3Δ5; pho5[GC, <TATA>]:UASP2mut	This study
yC24.1	yM1.12	matα; his3-11; his3-15; leu2-3; leu2-112; canR; ura3Δ5; pho5[GC, <TATA>]:UASP1mut	This study
yC132.1	YR22	mata; ade2-1; ura3-1; trp1-1; leu2-3,112; his3-11; can1-100; PHO4-MNase-3xHA KanMX6; PHO2Δ::URA3	This study
yC131.8	YR3	matα; his3-11; his3-15; leu2-3; leu2-112; canR; ura3Δ5; PHO5 Δ::URA3; PHO2-MNase-3xHA KanMX6	This study
yC138.6	yC131.8	matα; his3-11; his3-15; leu2-3; leu2-112; canR; ura3Δ5; PHO5::UASp1mut; PHO2-MNase-3xHA KanMX6	This study
yC139.5	yC131.8	matα; his3-11; his3-15; leu2-3; leu2-112; canR; ura3Δ5; PHO5[GC]::UASp2mut; PHO2-MNase-3xHA KanMX6	This study
yC159.3	yC131.8	matα; his3-11; his3-15; leu2-3; leu2-112; canR; ura3Δ5; PHO2-MNase-3xHA KanMX6	This study
yC162.1	yC159.3	matα; his3-11; his3-15; leu2-3; leu2-112; canR; ura3Δ5; PHO4Δ::URA3PHO2-MNase-3xHA KanMX6	This study

### Chromatin Endogenous Cleavage (ChEC)

Micrococcal nuclease-tagged strains were cultured at 30°C in 260 ml of YPAD to a final density of 3–4×10^7^ cells per ml. One hundred milliliters of culture were harvested for the zero hour time point. Cells from the remaining 155 ml of culture were washed with 20 ml of sterile water, resuspended in 420 ml of phosphate-free SCD medium and cultured at 30°C. ChEC was performed using cell extracts from 100 ml formaldehyde-cross-linked yeast cells as described previously [26] with the following exceptions: Formaldehyde was added to cultures at room temperature for 15 minutes. Cells were washed once with sterile water after harvesting. Protease inhibitors 4-(2-aminoethyl) benzenesulfonyl fluoride hydrochloride (AEBSF), Pepstatin A, and E-64 were added to all buffers at a final concentration of 2 µM. Complete Mini EDTA-free Protease Inhibitor Cocktail (Roche) was added prior to bead beating. The bead beating was performed on a MP FastPrep-24 machine (MPBio), set at 5.0 m/s for 2×20 seconds with a one minute break after the first 20 second beating.

After micrococcal nuclease cleavage reactions, samples were treated with RNase A at a final concentration of 100 mg/ml for 1 hour at 37°C. Incubation was continued for an additional hour after adding 1/40 volume of 10% SDS and 100 mg/ml Proteinase K. Reverse cross-linking was performed at 65°C overnight. One-third of the isolated DNA was digested with a restriction enzyme and separated in a 1.6% agarose gel in Tris-borate-EDTA at 4 V/cm for 4 hours and 30 min. Southern blotting and probe preparation was performed as previously described [29]. ^32^P-labeled *PHO5* upstream and open reading frame probes were prepared by random priming using a 500 bp NotI/BamHI fragment and a 687 bp ScaI/SalI fragment as template, respectively.

### Phosphatase Assay

Fifty microliters of cells were mixed with 250 µl 0.1 M pH 4.2 sodium acetate and 250 µl of 10 mg/ml 4-orthonitrophenylphosphate and incubated for 15 minutes at 37°C. Following incubation, 900 µl of 1.4 M sodium carbonate was added to the mixture and 800 µl of the final mixture was measured at optical density (OD) at 420 nm. The OD reading at 420 nm was divided by the OD at 600 nm to normalize for cell density.

### Topology Analysis

Analysis of topoisomer distributions was performed as previously described [29].

## References

[1] Lorch Y, LaPointe JW, Kornberg RD (1987). Nucleosomes inhibit the initiation of transcription but allow chain elongation with the displacement of histones.. Cell.

[2] Polach KJ, Widom J (1995). Mechanism of protein access to specific DNA sequences in chromatin: a dynamic equilibrium model for gene regulation.. J Mol Biol.

[3] Workman JL, Roeder RG (1987). Binding of transcription factor TFIID to the major late promoter during in vitro nucleosome assembly potentiates subsequent initiation by RNA polymerase II.. Cell.

[4] Kaplan N, Moore IK, Fondufe-Mittendorf Y, Gossett AJ, Tillo D (2009). The DNA-encoded nucleosome organization of a eukaryotic genome.. Nature.

[5] Mavrich TN, Ioshikhes IP, Venters BJ, Jiang C, Tomsho LP (2008). A barrier nucleosome model for statistical positioning of nucleosomes throughout the yeast genome.. Genome Res.

[6] Segal E, Fondufe-Mittendorf Y, Chen L, Thastrom A, Field Y (2006). A genomic code for nucleosome positioning.. Nature.

[7] Zhang Y, Moqtaderi Z, Rattner BP, Euskirchen G, Snyder M (2009). Intrinsic histone-DNA interactions are not the major determinant of nucleosome positions in vivo.. Nat Struct Mol Biol.

[8] Mao C, Brown CR, Falkovskaia E, Dong S, Hrabeta-Robinson E (2010). Quantitative analysis of the transcription control mechanism.. Mol Syst Biol.

[9] Pina B, Bruggemeier U, Beato M (1990). Nucleosome positioning modulates accessibility of regulatory proteins to the mouse mammary tumor virus promoter.. Cell.

[10] Li G, Levitus M, Bustamante C, Widom J (2005). Rapid spontaneous accessibility of nucleosomal DNA.. Nat Struct Mol Biol.

[11] Adams CC, Workman JL (1995). Binding of disparate transcriptional activators to nucleosomal DNA is inherently cooperative.. Mol Cell Biol.

[12] Bauer WR, Hayes JJ, White JH, Wolffe AP (1994). Nucleosome structural changes due to acetylation.. J Mol Biol.

[13] Norton VG, Imai BS, Yau P, Bradbury EM (1989). Histone acetylation reduces nucleosome core particle linking number change.. Cell.

[14] Eisfeld K, Candau R, Truss M, Beato M (1997). Binding of NF1 to the MMTV promoter in nucleosomes: influence of rotational phasing, translational positioning and histone H1.. Nucleic Acids Res.

[15] Svaren J, Horz W (1997). Transcription factors vs nucleosomes: regulation of the PHO5 promoter in yeast.. Trends Biochem Sci.

[16] Almer A, Rudolph H, Hinnen A, Horz W (1986). Removal of positioned nucleosomes from the yeast PHO5 promoter upon PHO5 induction releases additional upstream activating DNA elements.. Embo J.

[17] McAndrew PC, Svaren J, Martin SR, Horz W, Goding CR (1998). Requirements for chromatin modulation and transcription activation by the Pho4 acidic activation domain.. Mol Cell Biol.

[18] Griesenbeck J, Boeger H, Strattan JS, Kornberg RD (2003). Affinity purification of specific chromatin segments from chromosomal loci in yeast.. Mol Cell Biol.

[19] Venter U, Svaren J, Schmitz J, Schmid A, Horz W (1994). A nucleosome precludes binding of the transcription factor Pho4 in vivo to a critical target site in the PHO5 promoter.. Embo J.

[20] Carvin CD, Dhasarathy A, Friesenhahn LB, Jessen WJ, Kladde MP (2003). Targeted cytosine methylation for in vivo detection of protein-DNA interactions.. Proc Natl Acad Sci U S A.

[21] Adkins MW, Howar SR, Tyler JK (2004). Chromatin disassembly mediated by the histone chaperone Asf1 is essential for transcriptional activation of the yeast PHO5 and PHO8 genes.. Mol Cell.

[22] Adkins MW, Williams SK, Linger J, Tyler JK (2007). Chromatin disassembly from the PHO5 promoter is essential for the recruitment of the general transcription machinery and coactivators.. Mol Cell Biol.

[23] Ransom M, Williams SK, Dechassa ML, Das C, Linger J (2009). FACT and the proteasome promote promoter chromatin disassembly and transcriptional initiation.. J Biol Chem.

[24] Li B, Carey M, Workman JL (2007). The role of chromatin during transcription.. Cell.

[25] Kim HD, O'Shea EK (2008). A quantitative model of transcription factor-activated gene expression.. Nat Struct Mol Biol.

[26] Schmid M, Durussel T, Laemmli UK (2004). ChIC and ChEC; genomic mapping of chromatin proteins.. Mol Cell.

[27] Boeger H, Griesenbeck J, Kornberg RD (2008). Nucleosome retention and the stochastic nature of promoter chromatin remodeling for transcription.. Cell.

[28] Dhasarathy A, Kladde MP (2005). Promoter occupancy is a major determinant of chromatin remodeling enzyme requirements.. Mol Cell Biol.

[29] Boeger H, Griesenbeck J, Strattan JS, Kornberg RD (2003). Nucleosomes unfold completely at a transcriptionally active promoter.. Mol Cell.

[30] Fascher KD, Schmitz J, Horz W (1993). Structural and functional requirements for the chromatin transition at the PHO5 promoter in Saccharomyces cerevisiae upon PHO5 activation.. J Mol Biol.

[31] Barbaric S, Munsterkotter M, Goding C, Horz W (1998). Cooperative Pho2-Pho4 interactions at the PHO5 promoter are critical for binding of Pho4 to UASp1 and for efficient transactivation by Pho4 at UASp2.. Mol Cell Biol.

[32] Nourani A, Utley RT, Allard S, Cote J (2004). Recruitment of the NuA4 complex poises the PHO5 promoter for chromatin remodeling and activation.. Embo J.

[33] Luger K, Mader AW, Richmond RK, Sargent DF, Richmond TJ (1997). Crystal structure of the nucleosome core particle at 2.8 A resolution.. Nature.

[34] Shimizu T, Toumoto A, Ihara K, Shimizu M, Kyogoku Y (1997). Crystal structure of PHO4 bHLH domain-DNA complex: flanking base recognition.. Embo J.

[35] Rhodes D, Klug A (1980). Helical periodicity of DNA determined by enzyme digestion.. Nature.

[36] Fascher KD, Schmitz J, Horz W (1990). Role of trans-activating proteins in the generation of active chromatin at the PHO5 promoter in S. cerevisiae.. Embo J.

[37] Terrell AR, Wongwisansri S, Pilon JL, Laybourn PJ (2002). Reconstitution of nucleosome positioning, remodeling, histone acetylation, and transcriptional activation on the PHO5 promoter.. J Biol Chem.

[38] Han M, Kim UJ, Kayne P, Grunstein M (1988). Depletion of histone H4 and nucleosomes activates the PHO5 gene in Saccharomyces cerevisiae.. Embo J.

[39] Kaplan CD, Laprade L, Winston F (2003). Transcription elongation factors repress transcription initiation from cryptic sites.. Science.

[40] Merz K, Hondele M, Goetze H, Gmelch K, Stoeckl U (2008). Actively transcribed rRNA genes in S. cerevisiae are organized in a specialized chromatin associated with the high-mobility group protein Hmo1 and are largely devoid of histone molecules.. Genes Dev.

[41] Steger DJ, Haswell ES, Miller AL, Wente SR, O'Shea EK (2003). Regulation of chromatin remodeling by inositol polyphosphates.. Science.

[42] Feser J, Truong D, Das C, Carson JJ, Kieft J (2010). Elevated histone expression promotes life span extension.. Mol Cell.

[43] Zabaronick SR, Tyler JK (2005). The histone chaperone anti-silencing function 1 is a global regulator of transcription independent of passage through S phase.. Mol Cell Biol.

[44] Korber P, Barbaric S, Luckenbach T, Schmid A, Schermer UJ (2006). The histone chaperone Asf1 increases the rate of histone eviction at the yeast PHO5 and PHO8 promoters.. J Biol Chem.

[45] Magbanua JP, Ogawa N, Harashima S, Oshima Y (1997). The transcriptional activators of the PHO regulon, Pho4p and Pho2p, interact directly with each other and with components of the basal transcription machinery in Saccharomyces cerevisiae.. J Biochem.

[46] Wu WH, Hampsey M (1999). An activation-specific role for transcription factor TFIIB in vivo.. Proc Natl Acad Sci U S A.

